# Spatiotemporally and Sequentially-Controlled Drug Release from Polymer Gatekeeper–Hollow Silica Nanoparticles

**DOI:** 10.1038/srep46540

**Published:** 2017-04-24

**Authors:** L. Palanikumar, M. T. Jeena, Kibeom Kim, Jun Yong Oh, Chaekyu Kim, Myoung-Hwan Park, Ja-Hyoung Ryu

**Affiliations:** 1 Department of Chemistry, School of Natural Sciences, Ulsan National Institute of Science and Technology (UNIST), Ulsan 44919, Korea; 2 Department of Chemistry, Sahmyook University, Seoul, 01795, Korea

## Abstract

Combination chemotherapy has become the primary strategy against cancer multidrug resistance; however, accomplishing optimal pharmacokinetic delivery of multiple drugs is still challenging. Herein, we report a sequential combination drug delivery strategy exploiting a pH-triggerable and redox switch to release cargos from hollow silica nanoparticles in a spatiotemporal manner. This versatile system further enables a large loading efficiency for both hydrophobic and hydrophilic drugs inside the nanoparticles, followed by self-crosslinking with disulfide and diisopropylamine-functionalized polymers. In acidic tumour environments, the positive charge generated by the protonation of the diisopropylamine moiety facilitated the cellular uptake of the particles. Upon internalization, the acidic endosomal pH condition and intracellular glutathione regulated the sequential release of the drugs in a time-dependent manner, providing a promising therapeutic approach to overcoming drug resistance during cancer treatment.

Multidrug resistance (MDR) of cancer has been responsible for the high recurrence rate and failure of cancer chemotherapy; more than 90% of patients die due, to a certain extent, to MDR^1^,^2^,^3^,^4^,^5^,^6^. The primary strategy to overcome MDR is co-administration of multiple drugs^7^,^8^,^9^. However, varying drug uptake and suboptimal drug concentrations in heterogeneous tumour environments limit the synergistic efficacy of administered drugs^10^,^11^,^12^. Furthermore, combination therapy generally requires multiple injections, which may compromise patient compliance and complicate the therapy process. Therefore, sequential delivery of multiple drugs to the target site would be desirable for improving the efficacy of combination chemotherapy^13^,^14^,^15^,^16^.

Drug delivery systems using nanocarriers, such as polymeric and inorganic nanoparticles, are promising tools for providing sequential drug release in a one-tablet format owing to their tunable drug loading and release capabilities^17^,^18^,^19^,^20^. Recently, drug delivery systems based on core–shell nano- and microparticles have been reported to show sequential release kinetics through degradation of drug-loaded polymeric layers, with a potential to treat cancer^21^,^22^. However, achieving efficient sequential therapy with nanocarriers has been limited so far owing to the lack of an appropriate controlled-release system and possible leakage of encapsulated drugs during circulation^23^,^24^,^25^,^26^,^27^.

Hollow mesoporous silica nanoparticles (HMSNs) stand out as a promising solution to address challenges of a combination multidrug therapy because their void cores serve as extra reservoirs for drug storage^28^. Hydrophilic drugs can be loaded to the inner space of the hollow silica nanoparticles with a high loading efficiency, and hydrophobic drugs can be loaded on the mesoporous surface of HMSNs by physical adsorption^1^,^29^,^30^,^31^,^32^. Herein, we present a new class of drug delivery strategy for spatiotemporal release of different multiple drugs in a sequential manner by exploiting a pH- and redox-triggered release system using polymer-gated HMSNs (Fig. 1). Polymer gatekeepers, non-covalently attached to the surface of HMSNs, safely retain the cargos in storage until reaching the target site and successfully deliver the cargos into cancer cells via suitable release ways^33^,^34^. Furthermore, a stimuli-responsive charge reversal component was incorporated in HMSNs to accomplish programmable specific targeting of the tumour site. We applied cationic charge-generating polymer gatekeepers onto silica nanoparticles using a self-crosslinkable random copolymer containing pyridine disulfide (PDS), 2-(diisopropylamino) ethyl methacrylate (DPA), and polyethylene glycol (PEG). At physiological pH, the feature of the diisopropylamine moieties is initially to maintain a negative surface charge of the HMSN, minimizing the non-specific interactions with biomolecules. At the acidic pH of tumour environments, however, the protonated DPA consequently generates a positive surface charge on the HMSN that enhances the opportunity for tumour-targeted cellular uptake. Subsequently, upon internalization, a higher density of the cationic charge on the polymer shell is further generated at more acidic milieu of endosomes, resulting in the swelling of the polymer gatekeepers that induces the release of a hydrophilic drug, verapamil hydrochloride (Ver), and blocks the drug efflux pump P-glycoprotein which is the major protein for drug resistance^35^,^36^,^37^,^38^. Moreover, the positive charge facilitates the escape of HMSNs through membrane disruption^39^,^40^,^41^. In the cytosol, the polymer gatekeeper is expected to degrade through disulfide reduction by glutathione (GSH)^41^, causing a sequential release of a hydrophobic drug from inside the core to kill drug-resistant cancer cells. PEG on the surface of MSNs provides water solubility and prevents nonspecific interactions with biomacromolecules^41^,^42^,^43^.

In our study, two multidrug sets were used (Fig. 1), one with hydrophilic doxorubicin hydrochloride (Dox·HCl) and hydrophobic camptothecin (CPT) to understand the mechanisms of cellular interactions of the nanoparticles and their uptake, and another with hydrophilic Ver and hydrophobic doxorubicin (Dox, neutral form) to demonstrate the usability of the nanoparticles for combination multidrug therapy to overcome MDR. Similarly, paclitaxel (PTx) can be used, which is not protonated as a hydrophobic drug instead of Dox. Each drug was loaded into HMSNs without any chemical modifications, using a simple and robust method of combination of two different drugs.

## Results and Discussion

### Preparation of dual drug-loaded, dual stimuli- responsive PHMSNs

HMSNs were synthesized by a general template method according to previous reports^28^. Nitrogen adsorption-desorption isotherm measurements showed that the HMSN have a large Brunauer-Emmett-Teller (BET) surface area of 1307.6 m^2^/g, with a total pore volume of 1.56 cm^3^/g and a pore size of 2.4 nm (Figure S2). The HMSNs showed a uniform hollow structure to have the average diameter of 100 nm with shell thickness of 15 nm. We initially encapsulated two representative anticancer drugs; hydrophobic CPT and hydrophilic Dox·HCl with high loading capacities (Table 1). Unmodified pores of the HMSN shell with a large surface area can hold CPT by physical adsorption^29^,^30^,^31^,^32^, and Dox·HCl can be entrapped into the unmodified pores and hollow inner core^44^. Our previous work highlighted the importance of unmodified pores to hold an exceptionally large mass of a drug and to release it upon specific stimuli^33^,^34^. Consistent with the previous investigation, the Dox·HCl -loading efficiency up to 36 wt%, while the CPT-loading efficiency reached 80 wt%, with the proportional feeding ratio (Table 1). The cross-linked polymer layer was installed on the surface of the dual drug-loaded HMSN by the disulfide exchange reaction in the polymer shell which is coated by simple non-covalent electrostatic interactions between the negatively-charged HMSN and the positively-charged PEG–PDS–DPA copolymer (hereafter, abbreviated as PHMSN). The TEM image displayed a polymer-coating layer, which avoided agglomeration, and Dynamic light scattering (DLS) indicated an increase in the diameter of the nanoparticles from 100 to 130 nm (Fig. 2). Zeta potential measurements showed that the surface charge of the HMSNs was highly negative (−40 mV), but it became almost neutral (−2 mV) after the introduction of the polymer gatekeepers (Fig. 2B). In order to stably hold drugs in the HMSN pores, the coating polymers were further cross-linked by adding a partial amount of dithiothreitol (15 to 36 mol% of the PDS group), and the resulting crosslinking densities were 24 mol% and 53 mol%, respectively (Figure S3).

A pristine MSN has a limited capacity to serve as an ideal nanocarrier for drug delivery owing to unstable colloidal properties under physiological conditions, which may lead to undesired drug leakage before reaching the target^45^. To overcome these issues, polymer gatekeepers have been used in MSNs, which was shown to result in stable colloidal properties^33^,^34^. Consistently, no meaningful change in the PHMSN size was observed upon incubation with phosphate-buffered saline (PBS), pH 7.4, RPMI medium with 10% fetal bovine serum (FBS), and sodium acetate buffer, pH 5.5, for up to 72 h, indicating stable colloidal properties under physiological conditions, which may prevent undesired drug leakage before reaching target sites (Figure S3).

### Sequential drug release profile

**Figure 1:**
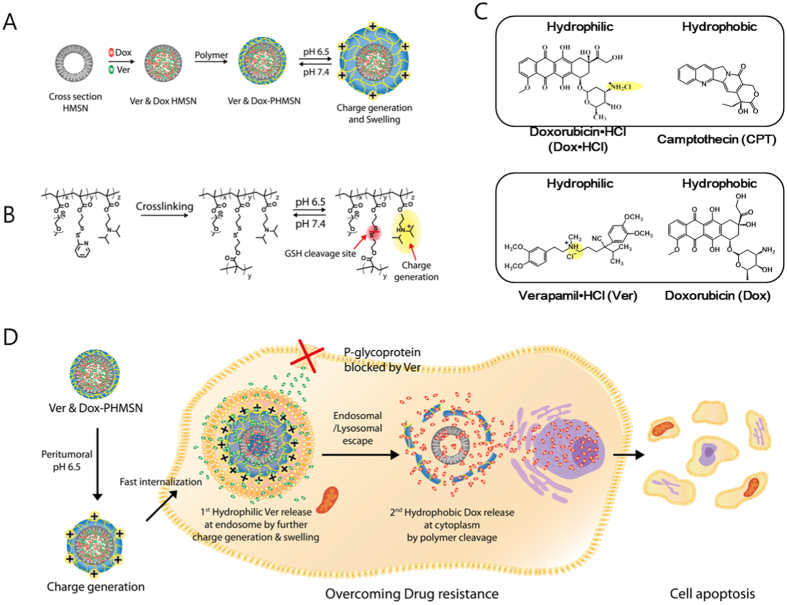
(**A**) Synthetic scheme for the preparation of a dual drug-loaded PHMSN using the polymer gatekeeper technique; (**B**) Disulfide cross-linking and pH-dependent cationic charge reversal by the protonation of the diisopropylamine group; (**C**) two combination sets of hydrophilic and hydrophobic drugs; (**D**) schematic illustration of the cellular uptake, endosomal escape, and GSH-mediated stimulus for sequential drug release. Under tumoural acidic conditions (pH 6.5), positive charge reversal results in a fast cellular uptake. Further increase of the positive charge of the polymer in the endosome (pH 5.0–5.5) induces the swelling of the polymer gatekeeper, followed by the release of hydrophilic Ver, and facilitates the endosomal escape of the nanoparticles. In the presence of intracellular GSH, the second drug, hydrophobic Dox, is released to kill the Dox-resistant cells.

**Figure 2:**
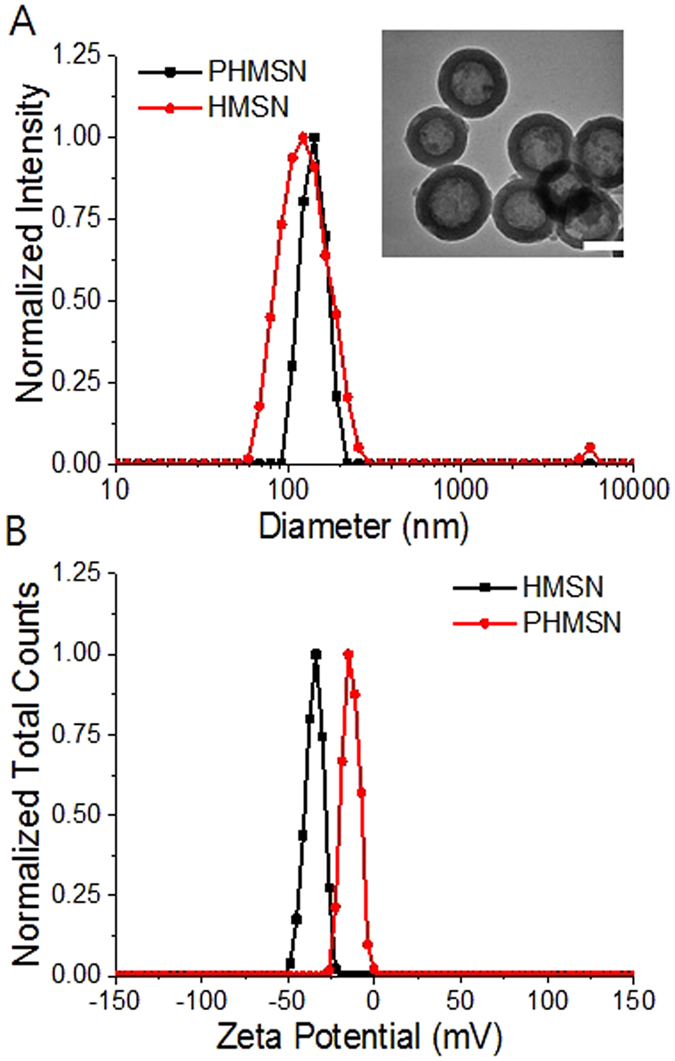
(**A**) Size analysis for HMSN and PHMSN (inset: TEM image of PHMSN) and (**B**) zeta potential measurement for HMSN and PHMSN. Scale bar represents 100 nm.

**Figure 3:**
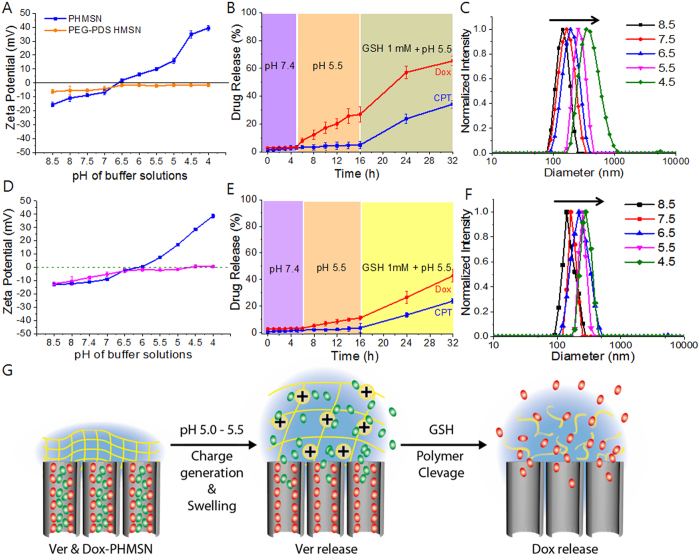
(**A**,**D**) Zeta potential measurement of PHMSNs and PEG–PDS-wrapped HMSNs in buffers with varying pH values for 24 mol% and 56 mol% crosslinked PHMSN. (**B**,**E**) CPT–Dox dual drug release profile of PHMSNs at different pH values for 24 mol% and 56 mol% crosslinked PHMSN. (**C**,**F**) Size analysis of PHMSN in buffer solutions with different pH values at 24 mol% and 56 mol% crosslinked PHMSN. (**G**) Schematic of charge reversal, volume change of polymer-shell and sequential drug release upon the change in pH and glutathione concentration.

It is important to retain the contents in a carrier at a physiological condition but selectively release them at a desired position. Nanoparticulate delivery system with pH responsiveness is one of the widely accepted routes. It is required that drugs are not or hardly released from a carrier in normal tissues and blood (pH ~7.4) but can be released in tumour tissues or even within cancer cells (pH 4 to 6.8)^35^,^36^,^37^,^38^. However, precisely maintaining drugs at a storage condition, without any leakage, and introducing them into a release medium are still challenging. In our system, the stable PHMSN with a covalently crosslinked polymer network possesses precise pH-responsive drug release characteristics. We used the PEG–PDS–DPA copolymer installed onto HMSNs, which can undergo charge reversal at acidic tumoural pH conditions, where DPA moieties are protonated to generate a positive charge owing to their pK_a_ values (Fig. 1). The pK_a_ value of the tertiary amine-containing polymer is around 6.7 at 24% crosslinking density and the unprotonated amine at physiological pH is neutral (Fig. 3A). The PHMSNs generated highly positive charges (+45 mV) at acidic pH conditions. At a neutral pH, however, they became negative (−5 mV), suggesting the successful preparation of charge conversion gatekeepers. No meaningful changes were observed in the case of control polymer-HMSN (PEG-PDS-HMSN) which has no DPA moiety.

To investigate the controlled sequential drug release behavior of Dox·HCl- and CPT-co-loaded PHMSNs, the nanoparticles were initially immersed in PBS at pH 7.4, and then the release medium was changed to an acetic acid buffer solution (pH 5.5) to stimulate an acidic environment. The system enables the drugs to be stably held at a neutral pH without any leakage (Fig. 3B). Remarkably, a high Dox·HCl release (>30%) was achieved at pH 5.5, but no CPT release was observed.

However, the use of a polymer network that is cross-linked by disulfide bonds enables the shell to be degraded upon the introduction of a reducing agent, ultimately releasing the second cargo. Following the Dox·HCl release, the addition of 1 mM GSH opened the gate by degrading disulfide bonds and triggering the release of CPT, over 40% within 24 h. Considering the release pattern, it is interesting to note that the release of Dox·HCl was controlled by the pH-responsive gatekeepers grafted on the surface of the HMSNs. The gatekeepers might form a dense layer on the surface that completely blocked the pores and thus prevented the release of Dox·HCl at pH 7.4. On the other hand, the gatekeepers become hydrophilic and swell rapidly owing to protonation at acidic conditions, resulting in loosening of the crosslinked networks and thus inducing the release of Dox·HCl (Fig. 3G). To demonstrate this phenomenon, we investigated the size change of the PHMSNs by varying pH of solutions. Surprisingly, a big increase in the size of the nanoparticles, from 130 nm (pH 7.5) to 300 nm (pH 4.5), was observed due to the protonation of the amine group at a low pH, which produces electrostatic repulsion between the quaternary amine moieties and therefore triggers the swelling (Fig. 3C).

When the crosslinking density increased to 53 mol%, the pK_a_ value of tertiary amine-containing polymer is around 6.2, the unprotonated amine at physiological pH is neutral (Fig. 3D) and the drug release is sustained and stabilized at pH 5.5 with Dox·HCl release over 15%. Similarly, reduced CPT release in the presence of GSH showed a remarkable change in contrast to the previous observation. In support, the swelling property at varying pH condition is controlled (Fig. 3F) reaching 280 nm (pH 4.5) from 130 nm (pH 7.5). Therefore, the proposed system can provide a spatiotemporal control over sequential release of drugs.

### Intracellular sequential and spatiotemporal drug release

**Figure 4:**
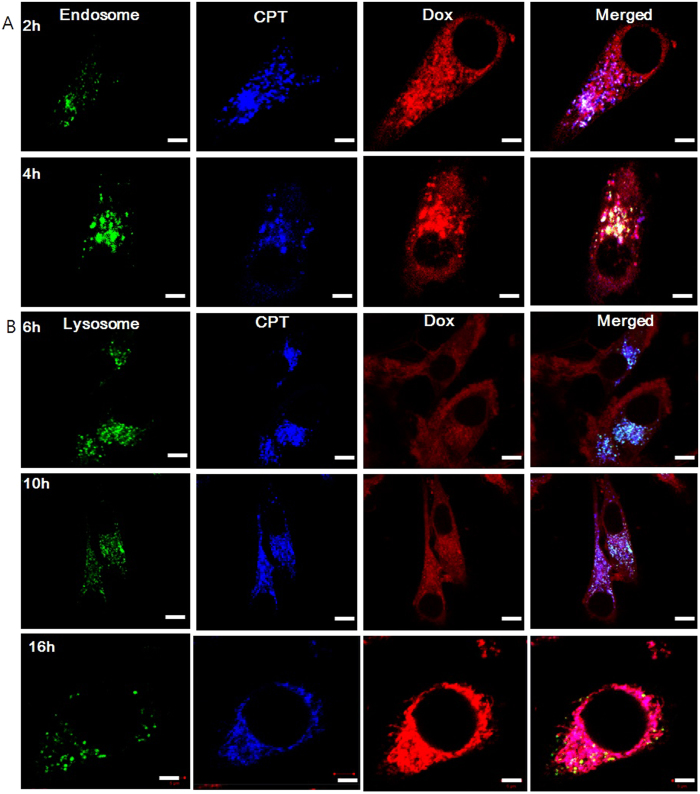
Confocal laser scanning microscope images of *in-vitro* cellular uptake for CPT & Dox·HCl loaded PHMSN in presence of (**A**) late endosome marker (green color) after 2 h and 4 h incubation. Blue and red color indicated the CPT and Dox, respectively. (**B**) Colozalization analysis in lysosomes with lysotracker green after 6 h, 10 h, and 16 h. Scale bar represents 10 and 5 μm, respectively. The cells were stained with late endosome.

To investigate the spatiotemporal property of the sequential drug release during the cellular uptake, the dual drug loaded PHMSN was incubated in human nasopharyngeal carcinoma cells (KB) and the intracellular co-localization was investigated by confocal laser scanning microscopy at different time points. Initially, the homogenous strong red (Dox·HCl) fluorescence was widely distributed in the cytoplasm, whereas the aggregated blue (CPT) fluorescence was observed, which suggests a successful pH-dependent release of Dox·HCl at the early period of endocytosis (Fig. 4 and S4). The acidic microenvironment of the endosome may facilitate the Dox·HCl release by swelling the polymer shell upon increasing the positive charge. The green fluorescence signal of late endosomal marker revealed that co-localization of Dox with late endosomes was not observed, whereas CPT was co-localized until 6 h. Similar to endosomes, Dox was not co-localized with green fluorescent lysosomes (Fig. 4B) from the initial time, and CPT co-localization was observed till 10 h incubation.

Whereas, no localization of CPT and lysosomal tracker could be seen after 10 h, suggesting the facilitation of escape and CPT release in the cytosol. Similarly, to confirm the subcellular colocalization of the delivered PHMSN, cells were morphologically examined using the TEM. After 20 h incubation, the majority of the nanoparticles were observed inside the lysosomes, fitting the literature reports average dimension 0.1 to 0.2 μm^46^,^47^,^48^. Consistent to the previous confocal microscopic observations (Fig. 4), the nanoparticles retained inside the lysosomes with their spherical morphology and revealed the feature of colocalization. Overall, the TEM images support the feature of colocalization of PHMSN in lysosomes and cytoplasm (Figure S5). These results revealed that the PHMSNs were capable of escaping or releasing long after entering the endosomal components.

### pH dependent cellular uptake analysis

**Figure 5:**
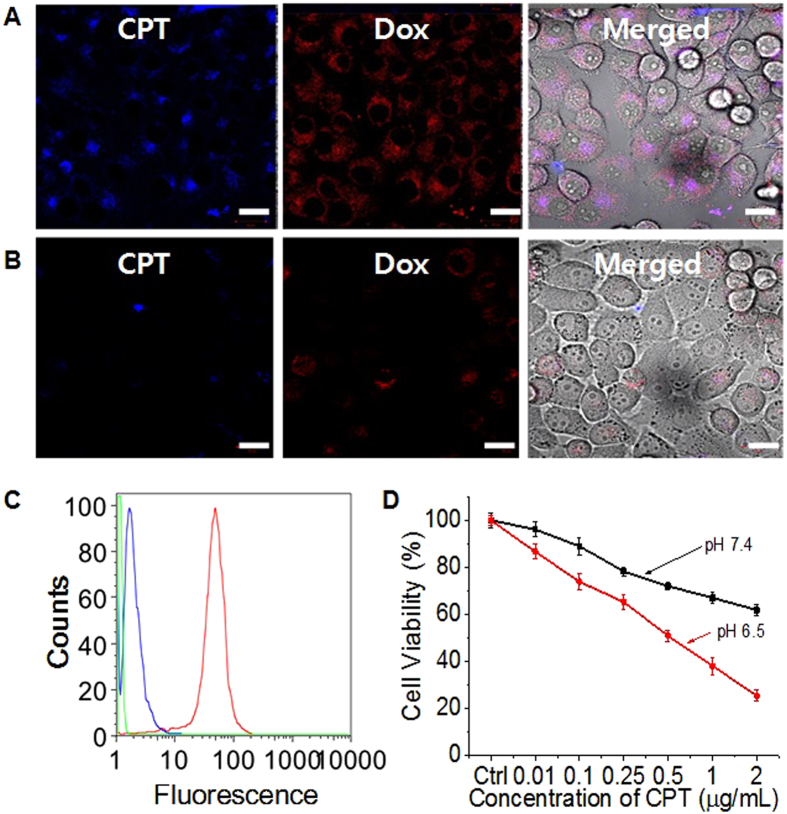
Images of *in vitro* cellular uptake of dual drug-loaded PHMSNs by KB cells after incubation at (**A**) pH 6.5 and (**B**) pH 7.4 DPBS. (**C**) Fluorescence-activated cell sorting analysis of cellular uptake of PHSMNs at pH 7.4 (red line), pH 6.5 (blue line), and in normal cell culture medium (green) after 2 h of incubation. (**D**) *In vitro* cytotoxicity analysis of KB cells incubated with dual drug-loaded PHMSNs at different pH, and (**E**) with pristine HMSN and PHMSN. Scale bar represents 20 μm.

The protonation event renders DPA positive, and facilitates its display on the surface for enhanced uptake by cancer cells. After 1 h of incubation at a peritumoural condition (pH 6.5), confocal microscope images of the PHMSNs exhibited a much higher cellular uptake compared to that of the same samples incubated at pH 7.4 (Fig. 5A and B). The obtained histograms by flow cytometry shown in Fig. 5C indicate that the interaction between the PHMSNs increased at pH 6.5 compared to the same nanoparticles at pH 7.4 as a function of pH-dependent uptake. The results revealed the uptake of the nanoparticles could be restricted at a neutral pH, whereas at a peritumoural condition pH 6.5, their high cellular uptake was efficiently carried out by the surface charge reversal. The toxicity of the nanocarrier incubated at the peritumoural pH condition was significantly higher than the value at the neutral pH (Fig. 5D). In view of the higher toxicity at peritumoural conditions, it would be clearly preferable to use the nanocarrier in cancer therapy due to its fast cellular uptake within a short period of incubation. Therefore, it is expected that a high dose of drugs was delivered inside the cancer cells after the incubation at the peritumoural pH condition. The nanoparticles had shown biocompatible behaviour in absence of drugs (Fig. 5E and S6).

### Mechanisms of cellular uptake analysis of PHMSN

**Figure 6:**
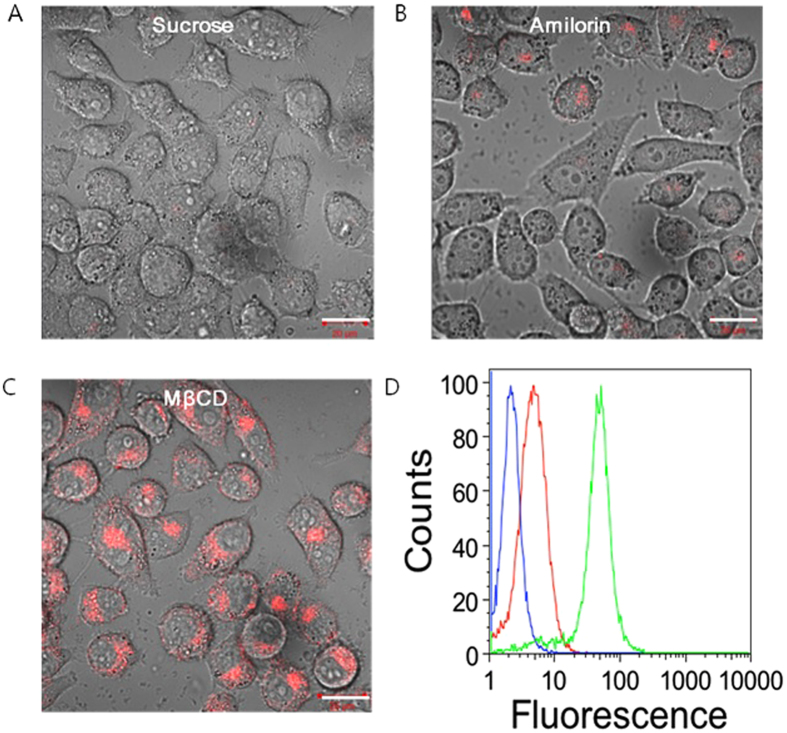
Confocal laser scanning microscope images of *in-vitro* cellular uptake for Dox-PHMSN in presence of (**A**)) sucrose (inhibitor for clathrin dependent endocytosis); (**B**) Amilorin (inhibitor for macropinocytosis dependent endocytosis), (**C**) MβCD (inhibitor for caveolae dependent endocytosis) after 2 h of incubation. (**D**) Flow cytometry analysis for Dox-PHMSN in KB cells after 3 h incubation. Scale bar represents 20 μm.

The cellular uptake of the PHMSNs was happened by clathrin- and macropinocytosis-mediated endocytosis (Fig. 6). The PHMSN uptake was inhibited in the presence of sucrose and amilorin, an inhibitor of clathrin-dependent uptake and macropinocytosis^42^,^43^. However, no inhibition was noted for methyl-β-cyclodextrin (MβCD), an inhibitor of caveolin-dependent uptake^42^,^43^.

Simultaneously, flow cytometry was further used to evaluate the uptake of the nanoparticles in the presence of inhibitors (Fig. 6D). The green (MβCD) fluorescence had a strong absorbance compared to that of the blue (sucrose) and red (amilorin) fluorescence. The results confirm the uptake of the PHMSNs by clathrin- and macropinocytosis-mediated endocytosis.

### Overcoming multidrug resistance by spatiotemporal drug release

**Figure 7:**
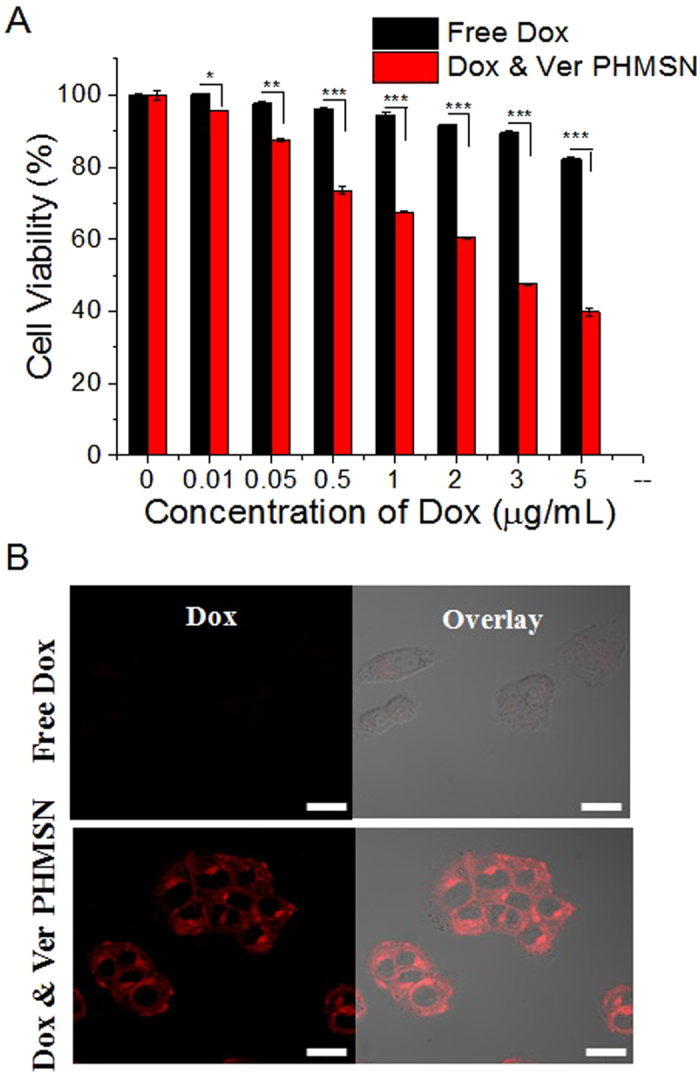
(**A**) Cell viability analysis for Free Dox; Ver & Dox coloaded PHMSN in Dox resistant MCF7/ADR (breast cancer) after 48 h incubation. (**B**) Confocal images to check the cellular uptake of Dox & Ver co-loaded PHMSN in MCF7/ADR cells after 2 h incubation. *P < 0.05, **P < 0.01, ***P < 0.001 compared to control, analysed by student’s t-test. Scale bar represents 20 μm.

Both the endosomal release by a pH change and the cytosomal release of drugs by the redox of GSH in a sequential manner indicate that the PHMSNs possess an excellent potential as a biologically important carrier for combination chemotherapy by co-administration of multiple drugs to overcome the MDR^49^,^50^,^51^,^52^,^53^. Overexpression of multidrug efflux pumps, such as P-glycoprotein and topoisomerase II, is conserved and mainly responsible for the drug resistance of cancer cells, leading to pumping drugs out through the plasma membrane, which decreases the concentration of drugs in the cytoplasm and causes lower anti-tumour effects^39^,^40^,^41^. Ver, a calcium channel antagonist, is a well-known P-glycoprotein inhibitor increasing the accumulation of anticancer drugs within cancer cells and reducing the clearance of Dox, which results in an increased plasma drug concentration and prolonged half-life^54^. To demonstrate the overcoming of drug resistance caused by overexpression of efflux pumps in cancer cells, we prepared Ver (hydrophilic drug)- and Dox (neutral form, hydrophobic drug)-co-loaded PHMSNs and analyzed their cellular uptake and cytotoxicity for P-glycoprotein-overexpressing breast cancer cell line, MCF7/ADR (Fig. 7). Importantly, the Ver- and Dox-co-loaded PHMSNs had a higher cytotoxicity against MCF-7/ADR cells compared to free Dox, with the IC_50_ value of 1 μg/mL for the Dox-loaded PHMSNs in the presence of the drug efflux inhibitor (Fig. 7A). The value is an excellent and exceptionally high inhibition concentration. The qualitative monitoring by confocal microscopic imaging also revealed that the Ver- and Dox-coloaded PHMSNs were taken up by the MCF-7/ADR cells and gradually released the Dox into the cells. The images in Fig. 7B and S7 show a significant increase in the Dox signal in the presence of Ver, whereas the fluorescent intensity of free Dox was very weak due to the drug efflux^55^. Therefore, we clearly confirmed that our synergistic multidrug delivery system increases the intracellular drug concentration at the peritumoural pH condition, enabling the drug delivery system to enhance the chemotherapeutic effect on Dox-resistant MCF-7/ADR cancer cells. Similar features were noted for MDK mediated drug resistance cells (Figure S8).

In summary, we developed a simple and robust sequential delivery platform by exploiting a pH-triggerable and redox-switched controlled-release system for combination therapy in cancer. We utilized the clinically relevant anticancer drugs to demonstrate the model of sequential combination therapy. The pH-responsive release of Ver and redox-sensitive release of Dox were achieved to enhance the therapeutic efficiency. Moreover, we reported a high loading efficiency for hydrophilic and hydrophobic dual drugs into unmodified pores of HMSNs for sequential and synergistic cancer treatment. Further research will focus on *in vivo* application of the novel sequential drug delivery system.

## Methods

### General

2-cyano-2-propyl benzodithioate (RAFT reagent), poly (ethylene glycol) methacrylate, 2-(Diisopropylamino)ethyl methacrylate, AIBN, tetraethyl orthosilicate (TEOS) and cetyl trimethylammonium bromide (CTAB) were purchased from Sigma Aldrich (Yongin, Korea) and TCI (Japan). Camptothecin was obtained from Ontario Chemicals Inc (Canada) and doxorubicin hydrochloride was obtained from Acorn-Pharma (U.S.A). Fetal bovine serum (FBS) was from Gibco, cell culture medium and reagents were obtained from Invitrogen (Korea). Cell viability analysis was measured using the alamarBlue^®^ cell viability reagent (DAL 1025, ThermoFisher, Korea) following the manufacturer’s protocol and analyzed using the fluorescence measurement from Tecan – Infinite 200 series reader. CellLight^®^ Late Endosomes-GFP, BacMam 2.0 (C10588) and LysoTracker^®^ Green DND-26 (L7526) were obtained from ThermoFisher (Korea). Unless otherwise stated, all used were obtained from commercial suppliers (Sigma Aldrich, TCI and Abcam) and were used as received. DLS measurements were made using a Malvern Nanozetasizer (Nano ZS series). UV-Visible spectra were measure using the JASCO V250 spectrophotometer. The fluorescence spectra were obtained using a JASCO FP-6500 spectrofluorimeter. PEG-PDS polymer was prepared and characterized using the 400 MHz, Bruker AVANCE III HD NMR spectroscopy following our previous literature^33^.

### Preparation of dual drug loaded PHMSNs

About 5 mg of HMSNs were added to 1 ml of DI water and sonicated to disperse the particles until a uniform colloidal solution was observed. To this solution, 2.5 mg, 5.0 mg and 7.5 mg of CPT were added (in DMSO), dissolved and allowed to stir for a period of 24 h in room temperature (RT). Then, dispersion was centrifuged to collect the CPT-loaded nanoparticles and kept the supernatant for calculating the drug loading content. In order to remove the DMSO, drug loaded nanoparticles were vaccum dried (under high vaccum), washed thrice with DI water to remove any DMSO and lyophilized again for further use. The mass of CPT loaded into the nanoparticles were calculated from the supernatant and washed supernatant solution. The amount of CPT loaded were calculated using the UV-Vis absorption spectra using a molar absorption coefficient of 42,282 M^−1^cm^−1^ with λ_max_ = 365 nm^34^. Then the nanoparticles dispersed in 1 mL of DI water under gentle vortexing, to this solution, 2.5 mg, 5.0 mg and 7.5 mg of Dox·HCl were added, dissolved and allowed to stir for a period of 24 h in RT. To this dual drug loaded nanoparticles in 1 mL aqueous suspension, 10 mg of PEG-PDS-DPA copolymer were added and stir for overnight. To crosslink the surface-wrapped polymer, a partial amount of DTT (15 mol% and 36 mol% against the PDS group) was added and the resulting solution was stirred for 3 h at room temperature. Drug-loaded PMSNs were collected by centrifugation and washed extensively with pH 7.4 phosphate buffer solution and distilled water. The crosslinking density was analyzed by checking the release of the byproduct pyridothione using the known molar extinction coefficient of pyridiothione (8.08 × 10^3^ M^−1^ cm^−1^ at 343 nm, Figure S3)^56^,^57^. Polymer wrapped, drug-loaded nanoparticles were washed twice with DI water and PBS buffer collected by centrifugation. The supernatant in the washing process was combined with the previous supernatant solution^33^. The mass of Dox·HCl loaded into MSNs (in weight %) was calculated by subtracting the mass of Dox·HCl in the supernatant from the total mass of drug in the initial solution. The amount of Dox·HCl adsorbed was analyzed by the UV-Vis spectroscopy using the molar excitation coefficient of 11,500 M^−1^cm^−1^. Drug loading was calculated using the following equation:


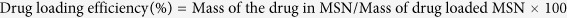



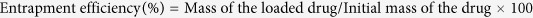


### Cell viability analysis in multidrug resistance cells (Pgp mediated resistance cells)

MCF-7/ADR cells were cultured (using DMEM medium) in sterile 96-well Nunc (Thermo Fisher Scientific Inc.) microtitre plate at a seeding density of 5 × 10^3^ cells/well and they were allowed to settle for 24 h under incubation at 37 °C and 5% CO_2_. In-order to check cell viability, the cells were then treated with different concentrations of Ver and Dox coloaded PHMSNs, (concentrations of 0.01, 0.05, 0.10. 0.25, 0.50, 1.00 and 2.00 μg/mL of Dox) were investigated after 48 h using the Alamar Blue assay with each data point measured in triplicate (initially, the nanoparticles were incubated with acidic pH 6.5 DPBS medium for a period of 2 h, washed replaced with fresh medium). Fluorescence measurements were made using the plate reader (Tecan Infinite Series, Germany) by setting the excitation wavelength at 565 nm and monitoring emission at 590 nm on the 96 well plates.

### Cell viability analysis in multidrug resistance cells (MDK resistance cells)

SNU-620-ADR/300 cells^55^ were cultured (using RPMI 1640 medium) in sterile 96-well Nunc (Thermo Fisher Scientific Inc.) microtitre plate at a seeding density of 5 × 10^3^ cells/well and they were allowed to settle for 24 h under incubation at 37 °C and 5% CO_2_. In-order to check cell viability, the cells were then treated with different concentrations of CPT and Dox coloaded PHMSNs, (concentrations of 0.01, 0.05, 0.10. 0.25, 0.50, 1.00 and 2.00 μg/mL of CPT) were investigated after 48 h using the Alamar Blue assay with each data point measured in triplicate (initially, the nanoparticles were incubated with acidic pH 6.5 DPBS medium for a period of 2 h, washed replaced with fresh medium). Fluorescence measurements were made using the plate reader (Tecan Infinite Series, Germany) by setting the excitation wavelength at 565 nm and monitoring emission at 590 nm on the 96 well plates.

## Additional Information

**How to cite this article:** Palanikumar, L. *et al*. Spatiotemporally and Sequentially-Controlled Drug Release from Polymer Gatekeeper–Hollow Silica Nanoparticles. *Sci. Rep.* **7**, 46540; doi: 10.1038/srep46540 (2017).

**Publisher's note:** Springer Nature remains neutral with regard to jurisdictional claims in published maps and institutional affiliations.

## Supplementary Material

Supplementary Information

## Figures and Tables

**Table 1:** Drug loading efficiency encapsulation efficiency analysis for dual drugs Dox + CPT in PHMSN.

Drug conc (Feeding)	Dox loading efficiency wt% (entrapment efficiency %)	CPT loading efficiency wt% (entrapment efficiency %)
2.5 mg/mL	13 wt% (30%)	18 wt% (46%)
5.0 mg/mL	24 wt% (32%)	37 wt% (60%)
7.5 mg/mL	26 wt% (24%)	44 wt% (54%)

*Drug loading efficiency (wt%) = Mass of drug loaded in nanoparticles/Mass of drug loaded nanoparticles.

Entrapment efficiency (%) = Mass of drug loaded in nanoparticles/Initial mass of drug.
