# Supplementary material for: Spatiotemporally and Sequentially-Controlled Drug Release from Polymer Gatekeeper–Hollow Silica Nanoparticles
Source: Sci Rep. 2017 Apr 24;7:46540. doi: 10.1038/srep46540 (PMC5402273; doi:10.1038/srep46540)
Supplement: Supplementary Information [file srep46540-s1.pdf]

Supporting Information

Spatiotemporally and Sequentially-Controlled Drug Release from Polymer Gatekeeper–Hollow Silica Nanoparticles

L. Palanikumar,[1] M. T. Jeena,[1] Kibeom Kim,[1] Joon Yong Oh,[1] Chaekyu Kim,[1] Myoung-Hwan Park,[2] and Ja-Hyoung Ryu[1]*

[1]Department of Chemistry, School of Natural Sciences, Ulsan National Institute of Science and Technology (UNIST), Ulsan 44919, Korea, [2]Department of Chemistry, Sahmyook University, Seoul, 01795, Korea.

E-mail: jhryu@unist.ac.kr

Experimental section

Synthesis and characterization of PEG-PDS-DPAMA copolymer.

A mixture of 2-cyano-2-propyl benzodithioate (RAFT reagent, 4.72 µL, 0.025 mmol), poly (ethylene glycol) methacrylate (1.02 g, 2.15 mmol), PDSEMA (549 mg, 2.15 mmol), 2-(Diisopropylamino)ethyl methacrylate (849.5 mg, 4.26 mmol) and AIBN (7 mg, 0.043 mmol) was dissolved in THF and then degassed by argon purge. The reaction vessel was sealed and then placed in a pre-heated oil bath at 70 ℃ for 12 h. In order to remove unreactive monomers and purify the polymer, the resultant mixture was precipitated in diethyl ether, which yields the random copolymer. ^{1}H NMR (400 MHz, Bruker AVANCE III HD, $CDCl_3$): δ 8.46, 7.68, 7.11, 4.21,4.07, 3.82, 3.55-3.52, 3.37, 2.97, 2.61, 1.85-1.86, 1.81. GPC (PMMA standard): Mn 78 kDa, Mw 132 kDa and PDI 1.69 (*Figure S1*).

Synthesis of HMSNs and characterisation. Ordered hallow mesoporous silica were prepared using the previously reported methods.[2] In detail, TEOS (10 mL) were added rapidly into the mixture of ethanol (430 mL), deionized water (DI, 60 mL) and ammonium aqueous solution (25–28%, 5 mL). Then the mixture was allowed to stirred at room temperature for 2 h, that result in the formation of white colloidal suspension of silica particles. These particles were centrifugally separated from the suspension and washed simultaneouly with deionized water and ethanol. About 100 mg of this silica nanoparticles was homogeneously dispersed in 10 mL of deionized water by ultrasonication. Then the solution were dispensed into the solution containing CTAB (150 mg), deionized water (30 mL), ethanol (30 mL), and ammonia solution (0.1 mL) under continous stirring for 30 min.

Immediately, TEOS (0.05 mL) was added. After 6 h, the obtained products were collected by centrifugation and redispersed in DI water (10 mL). Under vigorous stirring, 470 mg of Na_2CO_3 were added into the well-sonicated water suspension of the above solid SiO_2@CTAB/ SiO_2. After the reaction was stirred at 50℃ for 10 h, the products were collected by centrifugation and extensively washed with deionized water and ethanol. For the extraction process, the as prepared products were dispersed in 80 mL of acetone and refluxed at 80℃ for 48 h. The extraction was repeated three times to fully remove CTAB. The final products were collected and washed with DI water.

Drug release profile. The release kinetics of the drug-loaded nanoparticles in phosphate-buffered saline (pH 6.5) at room temperature were analyzed at excitation wavelengths of 480 nm and 360 nm for Dox and CPT, respectively, at several time points using a Shimadzu RFPC 5430 Spectrofluorometer. For Dox and CPT loaded PHMSN, drug release profile were analyzed using HPLC with methanol or acetonitrile and water. In order to measure triggered drug release profiles, 1 mM of GSH was added to the nanoparticle solution at 4 h and the release profile was analyzed using a fluorometer and HPLC (Agilent 1200 series).

Preparation of veramphamil and Dox coloaded PHMSN. HMSNs (5.0 mg) were dispersed well in 1 mL of hydrophobic doxorubicin drug solution, (2.5 mg in DCM) and stirred for a period of 24 h at room temperature. After the stipulated time periods, the drug-loaded nanoparticles were centrifuged and the supernatant was collected. The supernatant sample was used to measure the drug loading by collecting UV-Vis absorption spectra (V250 Jasco UV-Vis/NIR spectrophotometer). The drug-loaded nanoparticles were vacuum-dried, and redispersed in 1mL aqueous suspension containing 2.5 mg of veramphamil hydrochloride (Ver) and stirred for a period of 24h. To this solution PEG-PDS-DPA copolymer was added and stir for overnight. To crosslink the surface-wrapped polymer, a partial amount of DTT (20 mol% against the PDS group) was added and the resulting solution was stirred for 3 h at room temperature. Drug-loaded PMSNs were collected by centrifugation and washed extensively with pH 7.4 phosphate buffer solution and distilled water. HPLC analysis was used to measure the amount of Ver loading in the HMSN.[2] The Dox loading capacity was 21 wt%, while Ver loading capacity ranged at 9 wt% with a proportional ratio of 1:2.

Cell culture and viability analysis. Human nasopharyngeal carcinoma cells (KB) were cultured (using RPM 1640 medium) in sterile 96-well Nunc (Thermo Fisher Scientific Inc.) microtitre plate at a seeding density of 5 x 10^3 cells/well and they were allowed to settle for 24 h under incubation at 37 ℃ and 5% CO2. In-order to check cell viability, the cells were then treated with different concentrations of pristine HMSNs, and PEG-PDS-DPA capped HMSNs (25, 50, 75, 100 and 200 µg/mL) in KB cells. Dox-CPT-PHMSNs (concentrations of 0.01, 0.05, 0.10. 0.25, 0.50, 1.00 and 2.00 µg/mL of CPT) were investigated in KB cells. Initially, the nanoparticles were incubated with acidic (pH 6.5, Dulbecco's phosphate buffered saline, DPBS) and neutral (pH 7.4, DBPS) conditions for a period of 2 h, washed replaced with fresh medium, following previous reports.[7] Cell viability were measured at 48 h using Alamar blue assay with each data point measured in triplicate following manufacturer's protocol. In detail, fluorescence measurements were recorded after treating the alamar blue dye at each well, incubated until 4h at 37℃, viability was using the plate reader (Tecan Infinite Series, Germany) by setting the excitation wavelength at 565 nm and monitoring emission at 590 nm on the 96 well plates.

In-situ confocal microscopy for cellular internalization. KB cells were seeded in one well chmabered cover glass (Lab Tek II, Thermo Scientific) at a seeding density of 2 x 10^5 cells/well. After 24 h, cells were treated with Dox-CPT-PHMSNs nanoparticles at a final concentration of Dox/CPT at 10 µg/mL at pH 6.5 DPBS and pH 7.4 DPBS medium for a period of 2 h and then replaced with fresh medium.[7] To confirm the uptake, the fluorescent signal from Dox (approx. 559 nm emission) and CPT (approx. 410 nm emission) were observed and images were captured using the confocal microscope (Olympus FV1000 series). To check the endosomal and lysosomal escape/colocalization, the cells were pre-stained with the early endosomes-GFP and lysotracker green DND-26 by following the manufacturer's protocol. In order to check the endocytosis mediated uptake mechanism, cells were pre-incubated with inhibitors by previously reported methods.[8] The cellular uptake was monitored in the cover glass (Lab Tek II glass chamber cover glass, Thermo Fisher Scientific Inc) were monitored periodically using Olympus FV1000 confocal microscope connected to CO_2 incubator.

Flow-cytometry analysis. To analyze the cellular intake PHMSN nanoparticles at pH 6.5 and pH 7.4 DPBS medium, KB cells were seeded at 5 x 10^4 cells/well in 24 well plates. After

24 h, cells were treated with Dox-PHMSN nanoparticles at respective pH buffers and analyzed after 2 h incubation. Then the cellular uptake of doxorubicin was analyzed with FACS (FACS caliber E1377, BD Bioscience) using FL3H fluorescence filter (> 575 nm emission). Similarly for the cellular uptake pathway analysis, the cells were preincubated with the inhibitors and analyzed using the fluorescence from doxorubicin with FACS (FACS caliber E1377, BD Bioscience) using the FL3H fluorescence filter (> 575 nm emission). Sample and control pretreated adherent cell lines were washed twice with autoclaved 1x PBS and detached using the 0.25 % Trypsin-EDTA solution and immediately stored at ice cold condition for further use in FACS analysis. About 10,000 events were counted for each samples to analyze the uptake of Dox-PHMSN in KB cells in all the experiments.

In detail, for adherent cells the nanoparticle treated cells were washed twice with 1x PBS and detached using the 0.25 % Trypsin-EDTA solution, by following standard methods. Then the cells were collected by centrifugation and immediately stored in ice cold 1x PBS or 1x Annexin buffer for analyzing the cells in flow cytometry. Using the standard protocol from the instrument supplier, about 10,000 events were counted for all samples and compared the results with control. All data were computed using FACS-Jo software (BD Bioscience).

Statistical analysis. All statistical analysis was performed using the microsoft office excel 2010. All the experiments were repeated for three times with three replicates and checked for reproducibility. The results of cell viability analysis and drug release profile analysis were presented as mean ± SD (n=3).

Figures

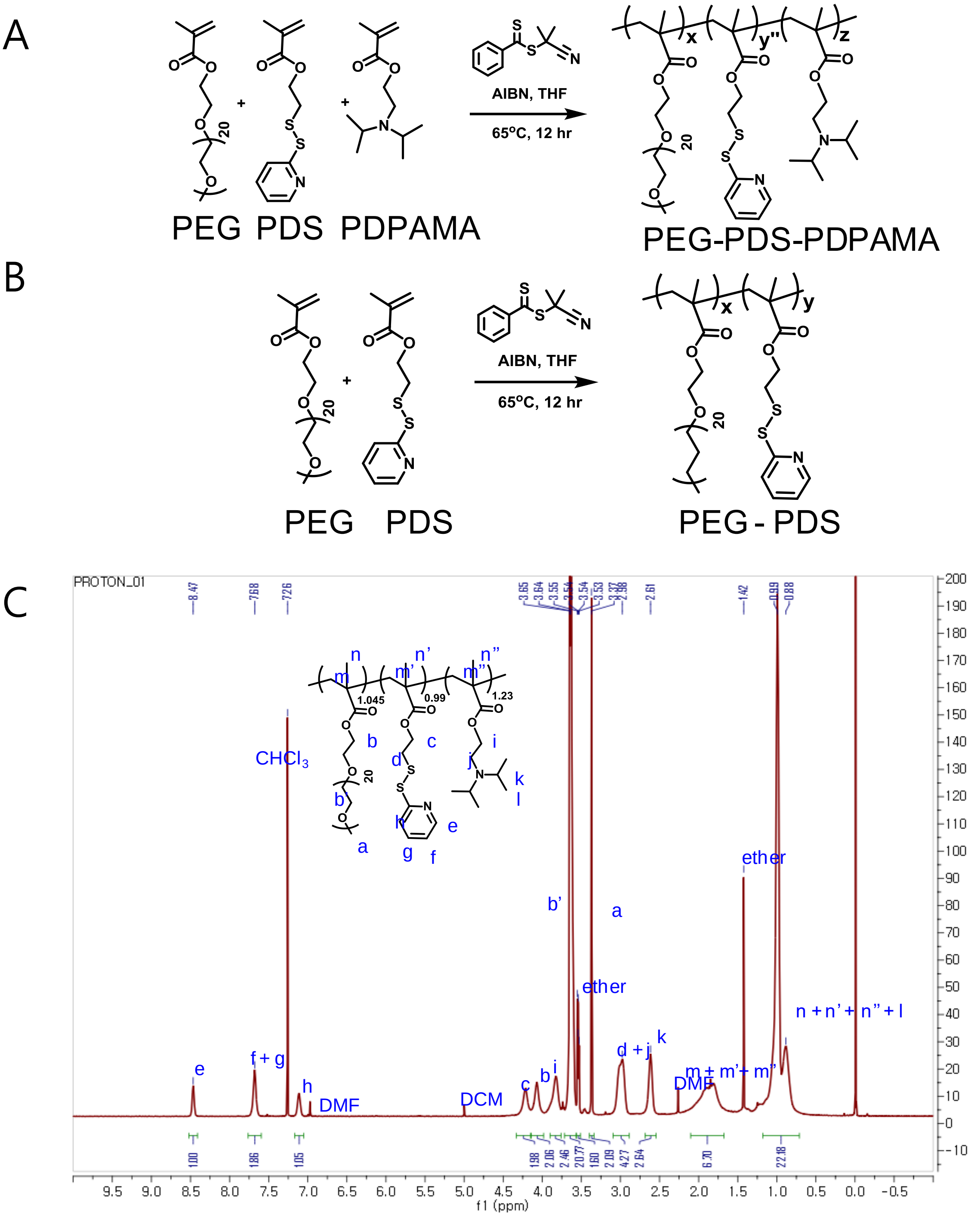

Figure S1. Synthetic scheme for the preparation of A) PEG-PDS-PDPAMA copolymer and B) PEG-PDS polymer. C) H^1 NMR characterization studies for PEG-PDS-PDPAMA copolymer

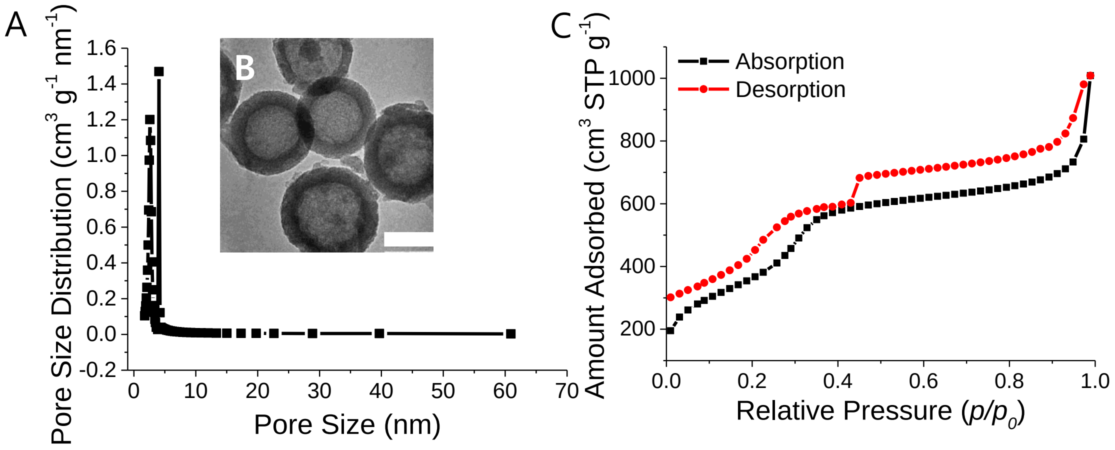

Figure S2. Characterization of HMSN A) BJH, B) TEM, C) BET analysis. Scale bar represents 100 nm.

Crosslinking density and colloidal stability analysis

In order to stably hold drugs in the HMSN pores, the coating polymers were further cross-linked by adding a partial amount of dithiothreitol (15 to 36 mol% of the PDS group), and the resulting crosslinking densities were 24 mol% and 53 mol%, respectively (Figure S3A and S3B). A pristine MSN has a limited capacity to serve as an ideal nanocarrier for drug delivery owing to unstable colloidal properties under physiological conditions, which may lead to undesired drug leakage before reaching the target.[9] To overcome these issues, polymer gatekeepers have been used in MSNs, which was shown to result in stable colloidal properties.[1] Consistently, no meaningful change in the PHMSN size was observed upon incubation with phosphate-buffered saline (PBS), pH 7.4, RPMI medium with 10% fetal bovine serum (FBS), and sodium acetate buffer, pH 5.5, for up to 72 h, a commonly used measurement period (Figure S3D, S3E and S3F).

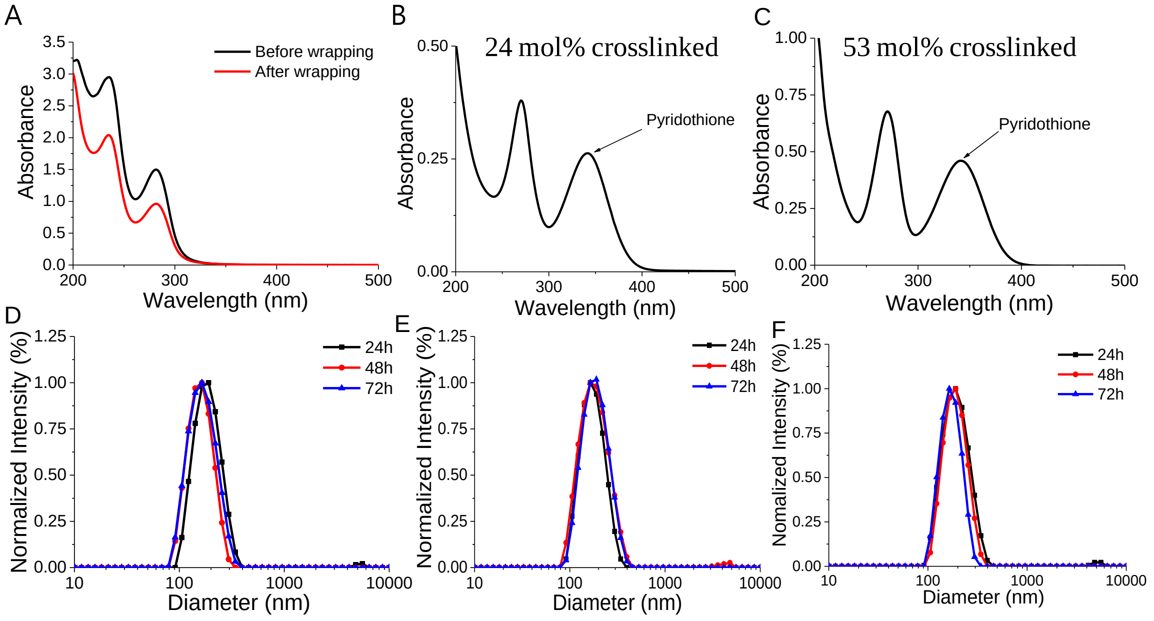

Figure S3. UV-Visible spectra to check the A) Concentration of polymer utilized to wrap the surface of HMSN B, C) UV-Visible spectra of byproduct pyridiothione by the addition of partial amount of DTT against 15 mol% and 36 mol% of PDS groups in PEG-PDS. Based upon mole % of PDS cleaved, crosslinking density was theoretically calculated by assuming that the formation of a single, cross-linking disulfide bond would require cleavage of two PDS units and produce two pyridiothione molecules. The known molar excitation coefficienct of pyridiothione was 8.08 x 10^3 M^{-1} cm^{-1} at 343 nm. Colloidal stability analysis for PHMSN D) at pH 5.5 sodium acetate buffer and E) pH 7.4 PBS F) RPMI 1640 medium with 10% FBS after 24 h, 48 h and 72 h**.**

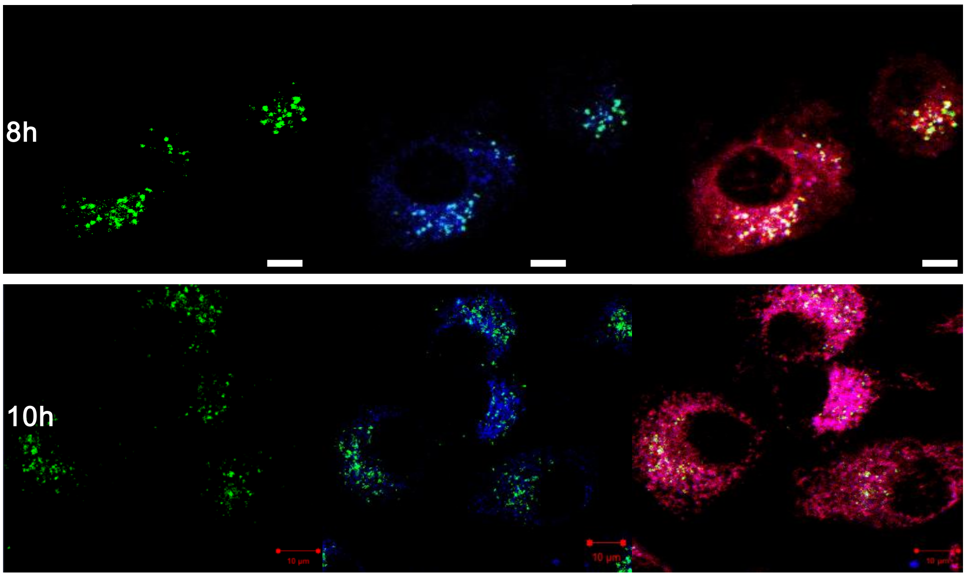

Figure S4. Confocal laser scanning microscope images of in-vitro cellular uptake for CPT & Dox•HCl loaded PHMSN in presence of A) late endosome marker (green color) after 8 h and 10 h incubation. Scale bar represents 10 µm

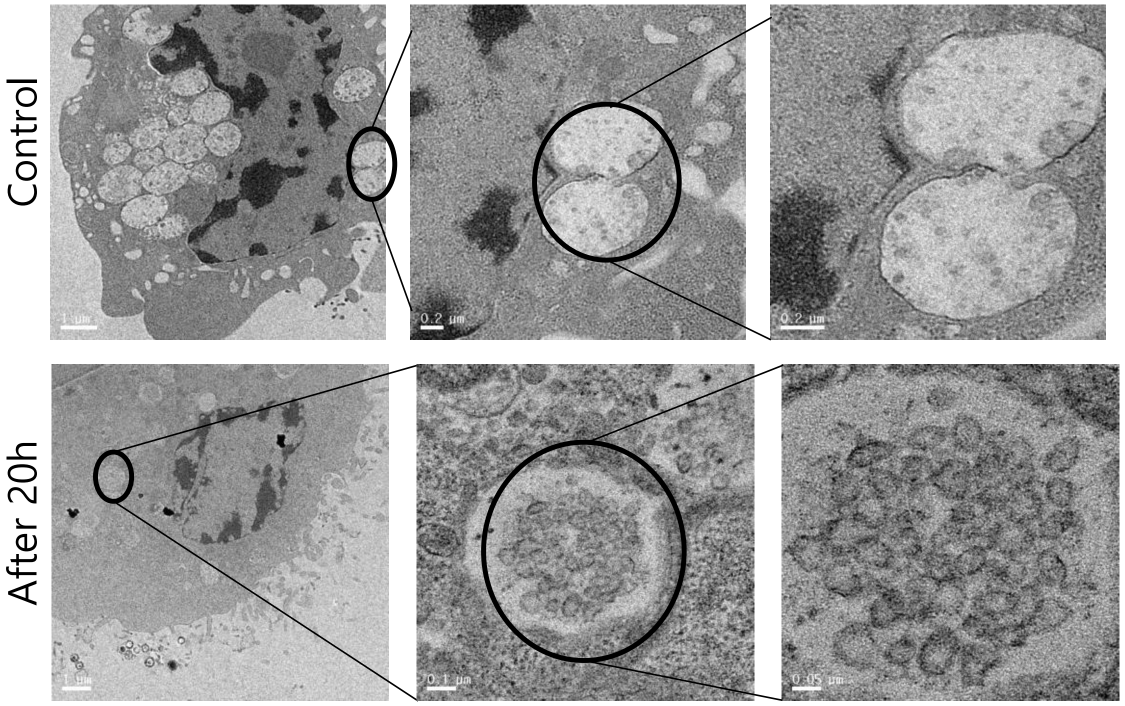

Figure S5. Cell TEM images of A) control KB cells and B) after 20 h treatment with PHMSN in KB cells. The magnified images show that the nanoparticles tent to release/escape from lysosomes to cytoplasm. TEM micrographs show intracellular PHMSN collect inside lysomal/endosomal and cytoplasm compartments over time. Scale bar represents 1 μm and 0.2 μm

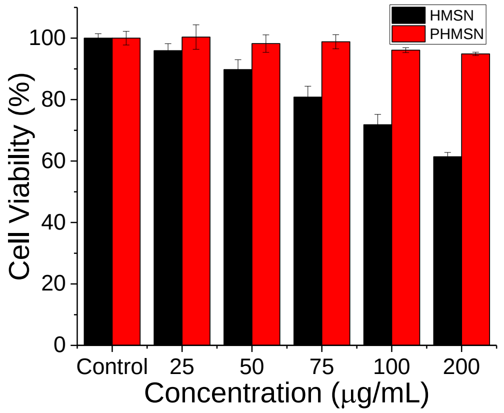

Figure S6. In vitro cytotoxicity analysis of KB cells with pristine HMSN and PHMSN after 48h incubation.

Analyzing the drug resistance in persence of free Ver.Hcl:

To analyze the significance of drug efflux inhibition in MCF7/ADR cells and as a positive control, the cell viability (24h incubation) for Dox & CPT coloaded PHMSN in presence and absence of free Ver•HCl results showed a significant change in cell viability in a stipulated

duration. Similarly, confocal microscope images shown a increased uptake of Dox in presence of free Ver•HCl treatment after 2h incubation (Figure S7).

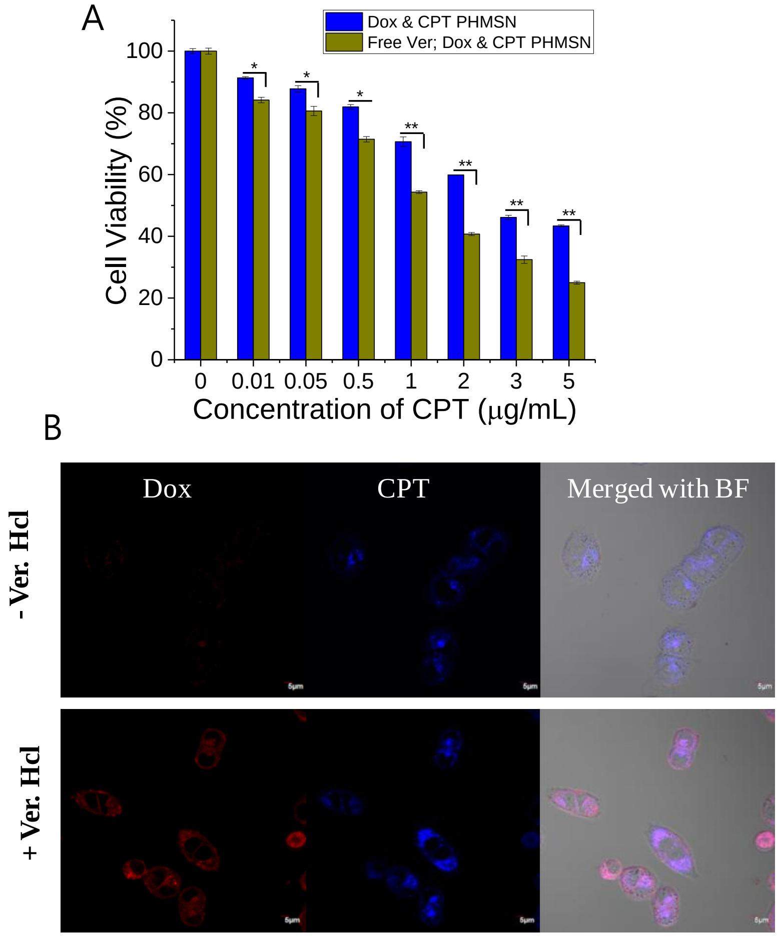

Figure S7. A) Cell viability analysis for Dox & CPT coloaded PHMSN in presence and absence of free Ver•HCl (5 µM) after 24h incubation. B) Confocal imaging to check the cellular uptake of Dox & CPT coloaded PHMSN in pH 6.5 buffer for 2h incubation. P < 0.05 * and 0.01 ** statistical significant when compared to their control and treatment groups. Scale bar in 5µm.

Cellular uptake analysis in MDK dependent drug resistant cells.

Overexpression of midkine (MDK), a cysteine-rich heparin-binding protein, in cancer cells contributes to drug resistance against Dox, 5-fluorouracil, and cisplatin in gastric cancer.[10] To evaluate the synergistic response and to overcome the Dox resistance in MDK-over expressing gastric cancer (SNU-0620-ADR/300) cells, Dox–HCl- and CPT-co-loaded PHMSNs were used. When the combination drugs were encapsulated into PHMSNs, a much enhanced cytotoxic effect was observed (Figure S8A). The half-maximum inhibition

concentration (IC_{50}) value of the Dox•HCl and CPT-loaded PHMSNs was as low as 1.2 μg/mL after 72 h of incubation, which is much lower than the values shown by single drug-loaded PHMSNs (Dox–HCl- or CPT-loaded PHMSNs), which were 4.2 and 2.1 μg/mL, respectively. The internalization was further confirmed by confocal microscopy analysis in a time-dependent manner (Figure S8B). As the time increased (from 2 h to 6 h), the Dox fluorescence signal was hardly observed, whereas the fluorescence of CPT was similarly maintained.

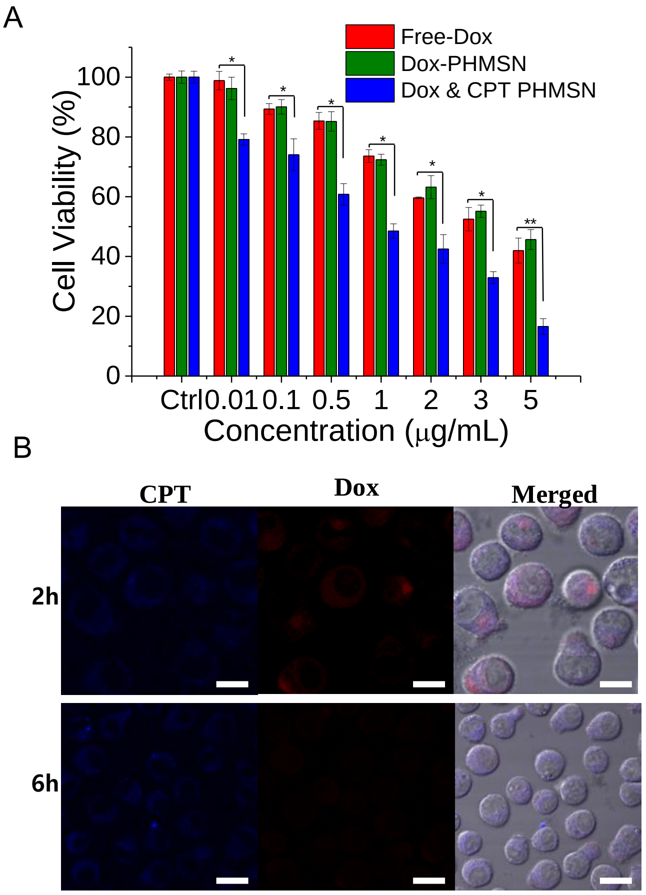

Figure S8. A) Cell viability analysis for Free Dox; Ver & Dox coloaded PHMSN in Dox resistant SNU-0620-ADR/300 (gastric cancer cells) after 48h incubation. B) Confocal images to check the cellular uptake of Dox & CPT co-loaded PHMSN in SNU-0620-ADR/300 cells after 2 h and 6 h incubation. * $P < 0.05$, ** $P < 0.01$, compared to control, analysed by student's t-test. Scale bar represents 20 μm.

Scrambling studies: To ensure the sequential release, one may hypothesise that the combination of Ver-Hcl (salt form as a hydrophobic drug) and Dox (neutral form as a hydrophobic drug) inside the nanoparticles may remain as the salt form and neutral form, that the proton will not simple scramble. If so, there is a possibility of concurrent release of these

two drugs and one can use PTx which is not protonated as a hydrophobic drug instead of Dox. In addition we tried to check the scrambling between Dox & Ver.HCl in test tube (in DI water). We hypothesized that the scrambling between Dox and Ver-HCl would increase the solubility of Dox due to the protonation. Initially hydrophobic Dox (2 mg) was dispersed in 1 mL of DI water and checked the solubility of hydrophobic Dox after 12 h in static condition by analyzing the absorbance. After this experiment, we performed the scrambling experiment. For this, Ver-HCl (2 mg) was added and allowed to stand for 12 h again. We performed this experiment in the simultaneously, to maintain the same Dox concentration. Then the supernatant solution was checked to analyze the scrambling by UV-visible spectroscopy. During this analysis, we observed that the concentration of Dox soluble in presence and absence of Ver.Hcl is negligible as shown **Figure S9**.

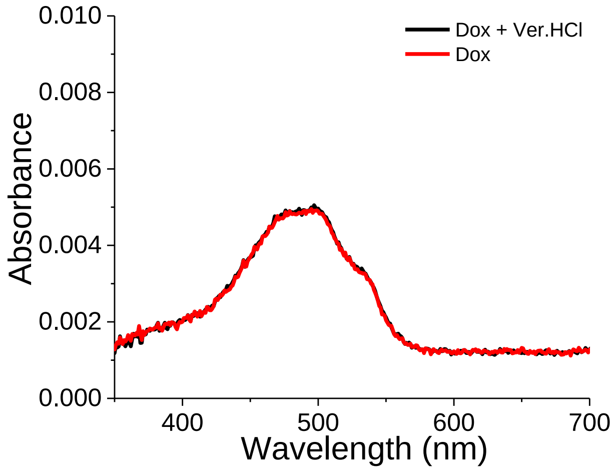

Figure S9. UV-visible absorbance of Dox before and after addition of Ver-HCl

As shown in Equation (1) and (2) the pK_a of Ver-HCl[11] is higher that Dox-HCl[12] because Ver contains tertiary amine while Dox contains primary amine.

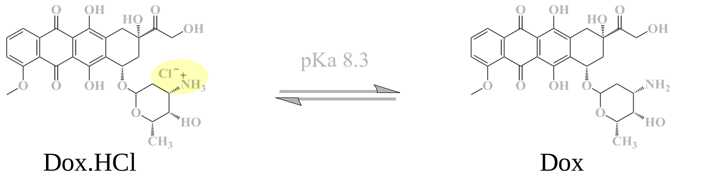

Eq (1)

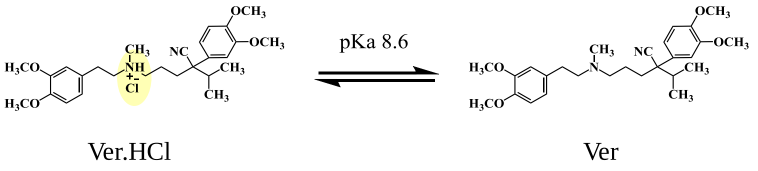

Eq (2)

Therefore, there is a very little possibility of scrambling of salt between Ver-HCl and Dox due to this pK_a difference. From our mixing experiment, we noted a negligible difference in absorbance in the Dox.

References

[1] Palanikumar, L., Choi, E. S., Cheon, J.Y., Joo, S.H. * Ryu, J.-H. *Adv Funct Mater* **25**, 957 (2015**)**.

[2] Fang, X., Chen, C., Liu, Z., Liu, P., & Zheng, N. *Nanoscale*,,**3**, 1632 (2011).

[3] Park, S *et al. Biomaterials* **31**, 7766 (2010).

[4] Ryu, J.-H. *et al.. J Am Chem Soc* **132**, 17227 (2010).

[5] Chang, B., Guo, J., Liu, C., Qian, J., Yang, W. *J Mater Chem.* 20, 9941 (2010).

[6] Lacroix, P. M., Graham, S. J. & Lovering, E. G. *J Pharma Biomed Anal* 9, 817 (1991).

[7] Kim, B.H., Yim, H., Jeon, Y.S., Na, K. & Hyeon, T. *J Am Chem Soc* **136**, 5647 (2014).

[8] Liang, L., Li, J., Li, Q., Huang, Q., Shi, J., Yan, H., Fan, C. *Angew Chem Int Ed* **53**, 7745 (2014)

[9] Firdessa, R., Oelschlaeger, T. A. & Moll, H. *Eur J Cell Biol* **93**, 323 (2014).

[10] Kang, H. C., Kim, I. J.. Park, J. H., Shin, Y., Ku, J. L., Jung, M. S., Yoo, B. C., Kim, H. K., Park, J. G. *Clin. Cancer. Res.* **10**, 272 (2004).

[11] https://pubchem.ncbi.nlm.nih.gov/compound/Verapamil_hydrochloride#section=Top

[12] Biomaterials for Cancer therapeutics: Diagnosis, prevention and therapy, edited by K.Park, *Woodhead Publishing Series in Biomaterials*, **66** (2013).
